# Editorial: Robotics in orthopedics and neurosurgery

**DOI:** 10.3389/frobt.2026.1982252

**Published:** 2026-10-20

**Authors:** Philipp Fürnstahl, Tom Vercauteren, Emmanuel Benjamin Vander Poorten, Ruixuan Li, Matthias Harders, Fabio Carrillo, Reza Monfaredi

**Affiliations:** 1 Balgrist University Hospital, Zürich, Switzerland; 2 School of Biomedical Engineering and Imaging Sciences, King’s College London, London, United Kingdom; 3 Department of Mechanical Engineering, KU Leuven, Leuven, Belgium; 4 Department of Computer Science, University of Innsbruck, Innsbruck, Austria; 5 OR-X Translational Center for Surgery, Balgrist University Hospital, University of Zürich, Zürich, Switzerland; 6 Children’s National Hospital, Sheikh Zayed Institute for Pediatric Surgical Innovation, Washington, DC, United States

**Keywords:** autonomy, human-robot collaboration, intraoperative imaging, minimal invasive surgery, real-time sensing, robot control, robotic perception

A major research challenge in surgical robotics is to enhance the capabilities, clinical efficacy, and efficiency of these systems, allowing deployment in increasingly complex and critical surgical tasks nearby fragile or critical structures in deformable and dynamic settings. This calls for better designs, better sensing approaches, more sophisticated algorithms, and advances in system intelligence and autonomy. While current robotic technologies augment human capabilities, they still depend heavily on direct human control. Achieving greater autonomy requires extensive research combining practical approaches and advanced robotic artificial intelligence (AI) techniques. Every autonomous decision based on real-time surgical data needs to uphold the highest standards in safety. In orthopedics and neurosurgery fields, robots need to deal both with soft, deformable tissue as well with hard, bone tissue. While operating in the vicinity of fragile and critical structures, they may need to exert large forces, thereby further complicating safety considerations. Maintaining surgical and functional accuracy beyond purely geometric accuracy in such dynamic environment remains an open challenge.

This Research Topic collects a selection of novel works featuring in-depth analyses, novel approaches, and innovative methodologies that contribute to the advancement of robotics in orthopedics and neurosurgery. A common denominator is the close integration of technical innovation with clearly identified unmet clinical needs, supported by a rigorous validation in synthetic or *ex-vivo* settings suggesting strong potential for establishing tangible real-world advancements in orthopedic and neurosurgical robot-assisted interventions.

The Research Topic starts by a mini-review by Cho et al. regarding human-centered assurance in robot-assisted orthopedics and neurosurgery. After summarizing different manners in which AI is being included in the next-generation robotic platforms, the work introduces ‘assurance frameworks’ that can help surgeons to effectively maintain oversight of complex AI-supported platforms. Human-centered assurance is argued as being a key enabler for safe adoption of increasingly autonomous surgical systems. Cho et al. proceed by sketching several essential research directions that could help in understanding how one can maintain human responsibility and control in the upcoming and increasingly autonomous orthopedic and neurosurgical interventions.

The paper by Davoodi et al. demonstrates the potential of robot-assisted surgery for pedicle screw placement (PSP) in the lumbar spine, focusing on an optical navigation and registration approach as an alternative to approaches that rely on intra-operative fluoroscopy and CT registration. Validated on both synthetic models and *ex-vivo* bovine spines, average position errors of respectively 2.61 mm and 1.51mm, and orientation errors of respectively 2.65° and 2.30° result. This demonstrates the potential of their approach to reduce radiation exposure of patients and clinicians. The authors conclude that although there are still challenges to address, robots equipped with optical tracking show considerable potential for clinical use.

While Davoodi et al. focus on lumbar spine fixation, Cong et al. extend robotic screw placement to cervical spine procedures. Cong et al. reported achieving 97.2% clinically acceptable robot-assisted placements of anterior transpedicular screws (ATPS) in the subaxial cervical spine using five human specimens. The study made use of the uOssa-Nav-s750 system (Wuhan United Imaging Surgical Co., Ltd., Wuhan, China), which is a robotic framework that employs 3D C-arm imaging, point-based rigid registration, and semi-automatic trajectory alignment. Once the robot is automatically aligned to the preplanned screw trajectory the surgeon performs the final fine adjustment and locking before drilling. The study on *ex-vivo* human specimens suggests that a robotic approach is feasible even for challenging interventions such as cervical ATPS, where current procedures are conducted manually. Despite demonstrated biomechanical advantages, ATPS is considered technically highly demanding today. Lin’s study showed that, provided further validation is conducted, robotic approaches may very well help broaden the adoption of ATPS in clinical settings in the future.

Inigo et al. focus on vertebroplasty, a minimally invasive procedure used to stabilize weakened or fractured vertebral bodies. In contrast to PSP where the goal is to accurately drill trajectories in which subsequently screws are placed, vertebroplasty requires direct needle insertion through the pedicle into the vertebral body in one go. Via the hollow channel of the needle, bone cement can then be injected to strengthen the fractured vertebral body. Again, in contrast to PSP, vertebroplasty typically cannot make use of pre-operative CT. To counter this lack of information, Inigo et al.’s Spine-DART system combines atlas priors with deep learning, differential rendering and a multi-view learned similarity loss ultimately requiring only two arbitrary X-ray images for 3D vertebral reconstruction and planning 3D transpedicular paths. This renders Spine-DART highly practical in real intraoperative settings. Trained on the colon and NMDID data-set, the method was tested on the VerSe-small and a single cadaver experiment, achieving competitive reconstruction results and plans that are considered clinically viable in the majority of cases.

Finally, in Gaytan et al. the authors describe the ability of the Robossis V2 system to support femur fraction reduction, by assisting in the re-alignment of the different femur bone fragments after fracture to restore anatomical position and promote healing. The paper describes the mechanical design and focusses on the improved workspace of Robossis V2 compared to its V1 predecessor, showing workspace coverages well beyond the clinically required alignment ranges. Further work on intuitive control and workflow integration is planned to optimize surgeon-robot collaboration in this high-force safety-critical application.

As illustrated across the works showcased in this Research Topic, advancing the capabilities of surgical robotics demands interdisciplinary research across multiple fronts. Aside from progress in actuation, sensing, and control, a key challenge is to improve the real-time understanding of the surgical scene to deal with deformation, physiological phenomena and uncertainty. The Research Topic showed that there is a major recent focus on developing new clinical workflows that exploit diverse real-time sensors and real-time data-processing to enable real-time, AI-empowered decision-making in complex surgical environments. As surgical robot capabilities improve and as robotic systems gain autonomy, ensuring their reliability through novel control and planning strategies is crucial for handling complex situations without compromising patient safety. Additionally, seamless data integration from visual and non-visual sources is essential for accurate decision-making. In parallel with increased autonomy, improvements in human-machine collaboration are needed to ensure robustness, safety and efficiency of operation. Beyond more intuitive interfaces that enhance human oversight, improved communication of intention among autonomous agents could strengthen human assurance. This, in turn, may facilitate smoother downstream transitions and support the subsequent regulatory processes and hence are critical to bring orthopedics and neurosurgical interventions to a new level of sophistication.

